# Hypothalamic tanycytes: potential roles in the control of feeding and energy balance

**DOI:** 10.1016/j.tins.2012.12.008

**Published:** 2013-02

**Authors:** Matei Bolborea, Nicholas Dale

**Affiliations:** School of Life Sciences, University of Warwick, Coventry, UK

**Keywords:** hypothalamus, tanycyte, energy balance, chemosensor, neural stem cell

## Abstract

Tanycytes, glial-like cells that line the third ventricle, are emerging as components of the hypothalamic networks that control body weight and energy balance. They contact the cerebrospinal fluid (CSF) and send processes that come into close contact with neurons in the arcuate and ventromedial hypothalamic nuclei. Tanycytes are glucosensitive and are able to respond to transmitters associated with arousal and the drive to feed. At least some tanycytes are stem cells and, in the median eminence, may be stimulated by diet to generate new neurons. The quest is on to understand how tanycytes detect and respond to changes in energy balance and how they communicate with the rest of the nervous system to effect their functions.

## Introduction

Several interconnected areas of the hypothalamus, including the arcuate nucleus, the ventromedial hypothalamic nucleus, and the paraventricular nucleus (PVN), form a neural network that controls the three factors that underlie energy balance: appetite and food intake, deposition of fat, and energy expenditure [1]. Physiological control of body weight by the brain requires the monitoring and integration of many signals that circulate in the body and provide information from the periphery (such as the stomach, pancreas, and fat stores). These key signals not only include circulating metabolites such as glucose, free fatty acids, and amino acids, but also specifically secreted hormones such as leptin, ghrelin, and insulin. Understanding how the hypothalamus responds to and integrates these signals is an essential part of finding ways to ameliorate the rising global incidence of obesity and related illnesses. Understandably, the major emphasis has been on elucidating the neuronal components of this network. As a result, other cell types present in the hypothalamus have received less attention.

**Figure 1:**
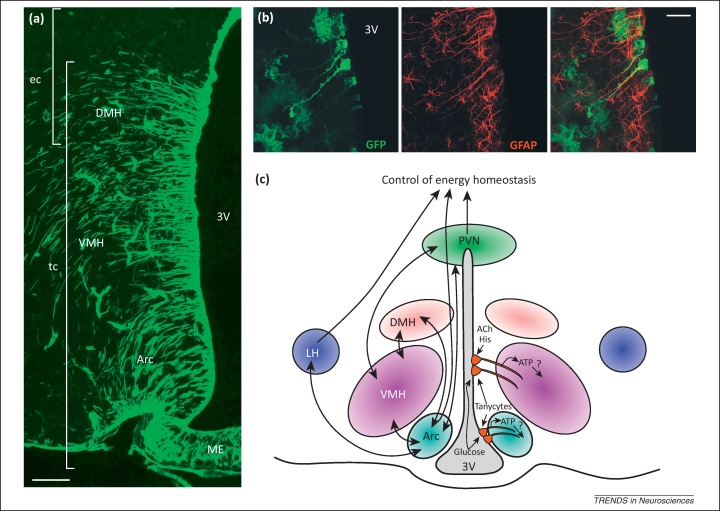
Hypothalamic tanycytes and their relationship to hypothalamic nuclei. **(a)** Immunostaining against vimentin (green) in the Djungarian hamster reveals the ependymal cells and tanycytes in the layer around the third ventricle (3V). Ependymal cells (ec) occur dorsally, whereas the tanycytes (tc) appear more ventrally – note their processes extending into the parenchyma, especially into the dorsomedial hypothalamus (DMH), ventromedial hypothalamus (VMH), and the arcuate nucleus (Arc) and median eminence (ME). Scale bar, 100 μm. **(b)** Detailed view of tanycytes genetically labeled in mouse with green fluorescent protein (GFP) and counterstained against glial fibrillary acidic protein (GFAP), a typical marker of glial cells [12]. Note the single process that terminates in a specialization. Scale bar, 20 μm. **(c)** Schematic diagram illustrating the hypothalamic nuclei involved in energy balance and the relationship of tanycytes to these nuclei. Abbreviations: ACh, acetylcholine; His, histamine; LH, lateral hypothalamus; PVN, paraventricular nucleus. Adapted, with permission, from [12] (b).

Many of the key nuclei controlling energy balance are in close proximity to the third ventricle (Figure 1a). The dorsal part of the lining of this ventricle contains a layer of ciliated ependymal cells. As the ventricle wall progresses more ventrally, tanycytes appear, initially interspersed with the ependymal cells, before progressively becoming more numerous and contiguous in the ventricle wall towards the median eminence (Figure 1). Ependymal cells are roughly cuboid, possess many beating cilia, and have only a very short process [2,3]. By contrast, tanycytes lack beating cilia, possess microvilli or other apical specializations, and have a long process that projects into the brain parenchyma [2–7] (Figure 1b).

The dorsal tanycytes near to the dorsomedial and ventromedial hypothalamic nuclei are often referred to as α1 and α2 tanycytes respectively, whereas those more ventral close to the arcuate nucleus and median eminence are referred to as β1 and β2 tanycytes, respectively [2]. β2 tanycytes differ from all other tanycytes because they have direct access to circulating plasma via fenestrations of the blood–brain barrier found at the median eminence [2]. Tanycyte processes can be several hundred microns in length. Regions of these processes are found in close association with capillaries and arterioles in the hypothalamus. The most fascinating aspect of the tanycytes is that their processes project into the very nuclei that control energy homeostasis (Figure 1c) and appear to come into close contact with the neurons of those nuclei [8,9]. The idea that tanycytes could somehow contribute to the control of energy homeostasis is, thus, irresistible. Although for a long time this idea has been purely speculative, emerging evidence supports the notion of tanycytes as chemosensory cells, diet-responsive adult neural stem cells, and the surprising loci for changes in gene expression related to the control of body weight. We review the evidence for these emerging roles and consider the extent to which these three functional roles could be linked.

## Tanycyte short-term signaling

Tanycytes are unlikely to signal via changes in membrane potential. They have very negative resting potentials, exhibit very low apparent whole-cell input resistances, and show no sign of spontaneous fluctuations in membrane potential [10]. Their membranes are highly selective for K^+^. Collectively these characteristics are similar to those of astrocytes and, like some astrocytes, tanycytes exhibit dye-coupling to each other [10].

Recent evidence suggests that tanycytes, similarly to various types of glial cells, signal via changes in intracellular Ca^2+^. Tanycytes respond to several transmitters, notably ATP, histamine, and acetylcholine (ACh), by exhibiting rapid and robust Ca^2+^ signals [11,12]. ATP acts via purinergic P2Y1 receptors to trigger Ca^2+^ signals that can propagate along the layer of tanycytes [11]. The speed of this Ca^2+^ wave is very similar to the propagation of Ca^2+^ signaling in other cell types such as radial glia and retinal pigment epithelial cells [13,14]. In addition, tanycytes are capable of releasing ATP [11]. It is highly likely that tanycytes signal as groups of cells, and that this signaling is coordinated via two mechanisms – at short ranges via gap-junction coupling between tanycytes and at longer ranges via the release and diffusion of ATP in the extracellular space with subsequent activation of P2 receptors on the tanycytes.

## Tanycytes as chemosensors

### The pancreatic β cell paradigm

**Figure 2:**
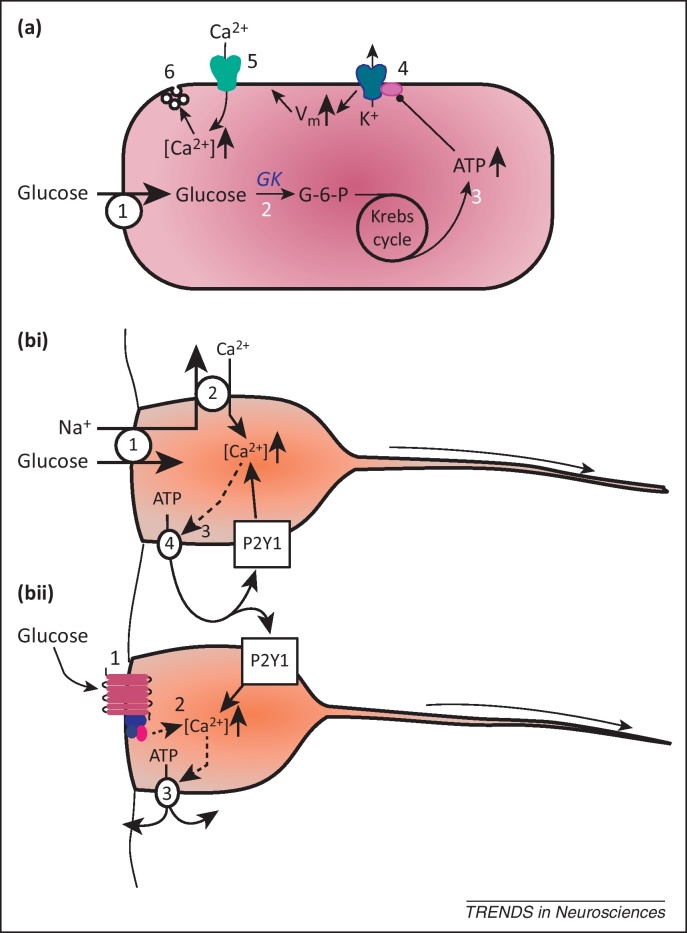
Hypotheses for glucose sensing in tanycytes. **(a)** The pancreatic β cell paradigm. Glucose is taken up into the cell (1), to be converted via glucokinase (GK) (2) to glucose-6-phosphate (G-6-P) which enters into the Krebs cycle. The net increase in ATP production (3) causes closure of the K-ATP channel (Kir6.2) (4) via a specific SUR subunit. This causes depolarization (V_m_), opening of Ca^2+^ channels (5), Ca^2+^ influx, and increased exocytosis (6). Most of these components are present in tanycytes, leading to the suggestion that this may be the mechanism by which tanycytes sense glucose. However, there are no reports of any voltage-gated channels in tanycytes. Furthermore, the ability of tanycytes to respond to non-metabolizable analogs of glucose has called this model into question. **(b)** Alternative models for tanycyte glucosensing. In the upper model (bi), glucose is taken up via a Na^+^-linked glucose transporter (1). The resulting Na^+^ accumulation and depolarization may reverse the Na^+^–Ca^2+^ exchanger (2), causing an increase in intracellular Ca^2+^. This increase may lead, via an unknown mechanism (3), to the release of ATP (4), which activates P2Y1 receptors resulting in a further increase in intracellular Ca^2+^ through mobilization of intracellular stores. The lower model (bii) is similar except that glucose and its analogs act via one or more G-protein-coupled receptors (1) to mobilize intracellular Ca^2+^ directly (2), which again leads to release of ATP (3) and activation of P2Y1 receptors. Modified, with permission, from [12].

The pancreatic β cell paradigm (Figure 2a) has been highly influential in glucosensing. In this model, glucose is transported into cells (usually via a facilitative glucose transporter) and converted to glucose-6-phosphate by glucokinase, an enzyme with a relatively low affinity for glucose that is within the physiologically important range of glucose concentrations (i.e., 4–10 mM). The glucose-6-phosphate enters the Krebs cycle, and subsequent metabolism results in an increase in intracellular ATP and the ratio of ATP to ADP. This in turn causes a K-ATP channel to close, which in the β cell comprises Kir6.2 and an associated sulphonylurea (SUR) subunit. Intriguingly, all of the necessary molecular components for this paradigm have been described in tanycytes, leading to the speculation that they may be glucosensors [2].

### Tanycytes respond to glucose

**Figure 3:**
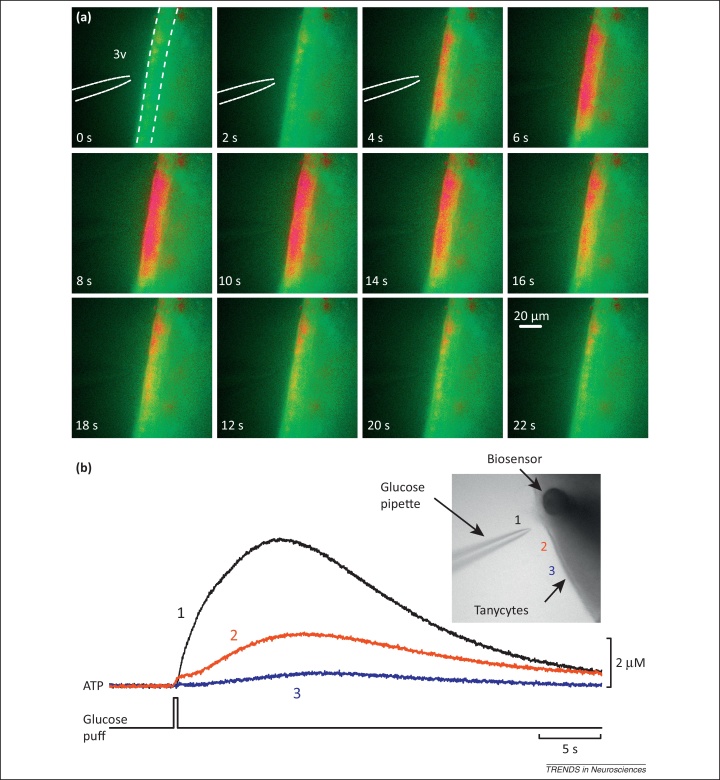
Glucose-sensing in tanycytes. **(a)** Fura-2 ratio images of acute hypothalamic slice from rats showing the increase in intracellular Ca^2+^ (denoted in red) evoked by a puff from a glucose-containing patch pipette (position indicated). This evoked a long-lasting Ca^2+^ wave (around 20 s in duration) in the tanycyte layer (delineated by dashed lines in top left image; note that the response started in the tanycyte layer and spread into the slice) [11]. **(b)** Recordings with an ATP biosensor of glucose-evoked ATP release from tanycytes. The inset shows recording arrangement and corresponding positions of the glucose pipette. A larger signal was observed when glucose was applied close to the biosensor (1) compared to when it was applied further away (2,3) [11]. 3v, third ventricle. Reproduced, with permission, from [11] (a,b).

Although direct evidence for the glucosensitivity of tanycytes has recently emerged via use of Ca^2+^ imaging, it does not wholly support the notion that tanycytes detect glucose via the β cell paradigm. In acutely prepared brain slices, tanycytes (α1, α2, β1) respond only rarely and weakly to changes of glucose concentration in the bathing medium. If they are first ‘conditioned’ by pretreatment with ACh and serotonin, small responses to changes in bath glucose can be observed [11]. However, the most prominent responses to glucose were apparent when glucose was applied selectively to their cell bodies via a puffer pipette [11] (Figure 3a). The glucose responses were accompanied by large Ca^2+^ waves, the release of ATP (Figure 3b), and activation of P2Y1 receptors. Interestingly, tanycytes also respond to non-metabolizable analogs of glucose [11]. One possibility is that tanycytes are designed to sense the composition of the CSF in the ventricle rather than the extracellular fluid in the parenchyma; CSF concentrations of glucose are likely to be higher than those in the parenchyma [15]. This possibility is reinforced by the observation that tight-junction proteins and functional tight junctions are present between tanycytes [16]. These observations suggest that responses to glucose in tanycytes must be mediated by a mechanism distinct from that of the pancreatic β cell paradigm. In this respect tanycytes have similarities to neurons in the lateral hypothalamus which also respond to non-metabolizable glucose analogs [17].

Independent confirmation of tanycyte responsiveness to glucose comes from studies of these cells in primary culture [18]. There are some differences from the observations made in brain slices because the cultured tanycytes appear to sense glucose in a manner that partially resembles the pancreatic β cell. Nevertheless, this study also reports the dependence on release of ATP (likely to be mediated via connexin hemichannel gating) and subsequent activation of P2Y1 receptors [18].

The molecular mechanisms by which tanycytes detect glucose and its non-metabolizable analogs remain unknown. One possibility is via the Na^+^-dependent glucose cotransporters (able to transport at least some analogs), which is the suggested mechanism for the orexinergic neurons of the lateral hypothalamus [17] (Figure 2bi). The glucose-linked influx of Na^+^ is proposed to reverse the Na^+^–Ca^2+^ exchanger and cause an increase in intracellular Ca^2+^. Alternatively, glucose and its analogs could bind to a G-protein-coupled receptor (GPCR) to trigger intracellular signaling (Figure 2bii). Although evidence suggests that tanycytes can respond to glucose via a novel ATP receptor-dependent mechanism, the pancreatic β cell paradigm could still have functional significance, given that its molecular components are expressed in tanycytes. The observations from cultured tanycytes are at least partly consistent with the β cell paradigm, and tanycytes could in principle exhibit multiple mechanisms of glucosensing. An intriguing idea is that the ability of tanycytes to integrate multiple types of input via Ca^2+^ signaling could trigger changes in gene expression within tanycytes, and regulate their propensity to divide and generate new cells within the hypothalamus.

## Regulated gene expression in tanycytes

Tanycytes express several important genes that have been linked to body weight and energy balance. Although tanycytes are not unique loci for expression of many of these genes, several lines of evidence suggests that their expression in tanycytes may be regulated in response to food restriction and, in seasonal mammals, by photoperiod, which is connected to natural adaptive changes in body weight (Box 1). The following examples highlight genes that are differentially regulated in tanycytes and start to link the function of tanycytes to the control of body weight and energy balance.

### The orphan GPCR: GPR50

GPR50 is a receptor that has sufficient homology to the melatonin receptors MT1 and MT2 for it to be considered as a third member of this receptor family although it does not bind melatonin. In rodents, GPR50 is expressed in many brain areas. In the hypothalamus, this receptor is mainly present in the lateral hypothalamus, the periventricular nucleus, the dorsomedial hypothalamus, and in tanycytes [19,20].

GPR50 is functionally linked to energy homeostasis, especially in the context of adaptive thermogenesis and torpor. GPR50 null mice are resistant to diet-induced obesity but also lose less weight when fasted [21]. Compared to wild type mice, GPR50 null mice exhibit a lower night-time body temperature despite displaying higher daily locomotor activity [21,22]. When fasted, GPR50 null mice – unlike wild type mice – reliably enter a state of torpor (as indicated by reduced body temperature and rate of oxygen consumption) [22]. The effects of GPR50 gene deletion appear to be mediated via thyrotropin-releasing hormone (TRH) and can be counteracted by injection of a TRH receptor agonist [22]. Although GPR50 expression in the dorsomedial hypothalamic nucleus seems to be important in mediating its actions, expression in tanycytes may also have a role. Under short photoperiods, there is a significant decrease in expression of GPR50 in the Djungarian hamster (*Phodopus sungorus*) [23], and this correlates with a greater tendency for these animals to display torpor under this state. Given the link between GPR50 and adaptive thermogenesis and torpor, this observation suggests that tanycyte-specific expression of GPR50 contributes to at least some of the actions of this receptor in the context of energy balance.

### Regulation of thyroid hormone signaling – deiodinase enzymes

Thyroid hormone signaling is a powerful regulator of energy balance and lipid metabolism [24,25]. Although the peripheral effects of thyroid hormone are well known, recent evidence suggests that the actions of thyroid hormone in the hypothalamus are also important and require tanycytes [8,26].

Thyroid hormone is initially synthesized as a prohormone, L-thyroxine (3,3′,5,5′-tetraiodo-L-thyronine), or T_4_. This prohormone must be converted to the active hormone, triiodothyronine (3,5,3′-triiodo-L-thyronine) or T_3_. Inactivation of thyroid hormone signaling occurs through further deiodination to generate the inactive T_2_. There are three deiodinase enzymes, Dio1–3, which have differing characteristics and are essential for both the conversion of T_4_ to T_3_ and the inactivation of T_3_ to T_2_ [27]. Both Dio1 and 2 can convert T_4_ to T_3_; however, in the brain Dio2 is the predominant activator of this signaling pathway. By contrast, Dio3 converts T_3_ to T_2_ and hence inactivates thyroid hormone signaling.

Tanycytes (α and β) are the main locus for expression of Dio2 in the rodent brain [8,28,29]. They also express the organic anion transporting polypeptide 1C1 (OATP1C1) and monocarboxylate transporter 8 (MCT8), which transport T_4_ and T_3_ [22,30,31]. Via MCT8 and OATP1C1, the prohormone T_4_ can be taken up by tanycytes from the circulation. The deiodinases then convert T_4_ to the active T_3_, which can diffuse into the surrounding hypothalamic nuclei. Tanycytes can thus be considered as the gatekeeper for access of thyroid hormone into the hypothalamus [32]. Interestingly, fasting will upregulate the expression of both Dio2 and MCT8, potentially allowing greater central levels of T_3_ [8,22]. The upregulation of Dio2 and consequent increased local production of T_3_ in the hypothalamus is very important in regulating the responses of neuropeptide Y (NPY)-containing neurons in the arcuate nucleus to food deprivation [8].

Axons from TRH-containing neurons of the PVN project into the median eminence where the neuron terminals make contact with the β2 tanycytic end feet [33,34]. Release of TRH from these endings is ultimately an important regulator of thyroid hormone (T_4_) secretion. The β2 tanycytes express pyroglutamyl peptidase II (PPII) which specifically degrades TRH [33,34]. The interactions between the β2 tanycytes, the terminals of the TRH-containing neurons, and the expression level of PPII determine the amount of circulating TRH and hence T_4_ secretion from the thyroid. Interestingly, the expression of PPII in the tanycytes is upregulated by the amount by T_3_, completing a classical feedback loop that controls the circulating levels of TRH and ultimately T_4_ [33,34].

One of the most profound changes in seasonal rodents occurs in the expression of the Dio2 genes. In numerous seasonal species studied, T_3_ levels decrease under short photoperiod, and this is mediated through downregulation of Dio2 and upregulation Dio3 in tanycytes [31,35–37]. This gene regulation and its consequences for the central actions of T_3_ is functionally important because T_3_ hypothalamic implants inhibit the normal loss of weight and catabolism of fat depots that occurs under short photoperiod [30,38,39].

### Neuromedin U receptor

Neuromedin U (NMU) is a neuropeptide linked to energy balance [40]. Intracerebral injection of NMU decreases food intake, and in obese models increases physical activity, energy expenditure, and thermogenesis. NMU null mice exhibit hyperphagia, increased body weight, and reduced energy expenditure [40]. NMU acts via NMU-receptor 2, and this receptor is expressed in tanycytes [41]. Although this receptor is also expressed in the arcuate and dorsomedial hypothalamic nuclei, the functional importance of tanycytic expression is suggested by the fact that expression of this receptor is upregulated specifically in tanycytes in F344 rats under long photoperiod [42] (see Figure I in Box 1).

### Retinoic acid signaling

Recent evidence has shown the regulation of retinoic acid signaling in the tanycytes of photoperiodic rats [43,44]. The expression of transthyretin (TTR), a transporter for vitamin A and its metabolite retinoic acid, is downregulated under short photoperiod in the tanycytes of F344 rats [42]. Other genes (transporters, receptors, and enzymes) involved in the retinoic acid signaling pathway display seasonal alterations of expression in tanycytes [23,42,45]. For example, mRNAs in tanycytes for the receptor for retinol-binding protein (RBP), STRA6, and cellular RBP-1 (CRBP1), as well as an enzyme that converts vitamin A to retinoic acid, retinaldehyde dehydrogenase 1 (RALDH1), are decreased by almost twofold when animals are under a lean short photoperiod state [42] (see Figure I in Box 1). This decrease in retinoic acid signaling under a short photoperiod is potentially highly significant because retinoic acid regulates the ability of tanycytes to proliferate and generate new cells in the hypothalamus (discussed below).

## Tanycytes as adult neural stem cells

Neural stem cells are defined by their capacity to self-renew and to generate cells other than themselves through asymmetric cell division [46,47]. Two areas of the adult brain, the subventricular zone of the lateral ventricle and the dentate gyrus of the hippocampus, contain neural stem cells. New evidence suggests that there are also neural stem cells in the adult hypothalamus. The identity of these cells is not completely clear because they are distributed both in the hypothalamic parenchyma and in the ependymal layer (Table 1), but tanycytes (both α and β) are an important – although not exclusive – component of this stem-cell population.

Morphologically, tanycytes resemble radial glial cells. During development of the cortex radial glial cells are neural progenitors and form a scaffold for the migration of newly generated neurons [48]. Radial glial cells release ATP, which controls neural proliferation [13]. Indeed, ATP regulates progenitor-cell proliferation in several other contexts including the developing retina, the cochlea, and the subventricular zone. In these cases, ATP acts via P2Y1 receptors to stimulate Ca^2+^ waves in the progenitor cells [13,14,49]. Expression of particular types of ectonucleotidase that degrade extracellular ATP (such as ectonucleoside triphosphate diphosphohydrolase 2, NTPDase2) are associated with these stem-cell populations [50,51]. Tanycytes possess some of the signaling systems associated with stem cells: they can release ATP, possess P2Y1 receptors, express NTPDase2 [50], and respond to activation of these receptors with vigorous Ca^2+^ waves [11]. In rodents, as well as in humans, tanycytes express nestin [23,52,53], vimentin [19,54,55], and doublecortin-like [56] proteins associated with neural precursor cells. Two recent papers demonstrate that at least some tanycytes express Sox2 [57,58], a marker of neural stem cells in the subventricular zone and dentate gyrus [47,59,60].

### Proliferation

The use of bromodeoxyuridine (BrdU), which is incorporated into the DNA of dividing cells, reveals that tanycytes (both α and β) divide, a necessary but not sufficient characteristic of putative stem cells (Table 1). They also transiently express cell-cycle proteins such as Ki67. Although tanycytes divide, they are not unique in this attribute; other cell types in the hypothalamic parenchyma also proliferate [58,61–63]. Interestingly, in photoperiodic mammals, tanycyte proliferation is stimulated by short and inhibited by long photoperiods (discussed further below). Because tanycytes have physiological functions, their proliferation could itself be functionally significant quite apart from any subsequent differentiation of the daughter cells into other hypothalamic cell types.

### Differentiation and multipotency

If tanycytes really are neural stem cells, they must be able to generate other cell types in the hypothalamus. In one of the first studies to address this problem, an adenoviral green fluorescent protein (GFP) construct was injected into the third ventricle to label tanycytes and ependymal cells [64]. Subsequently, a small number of GFP-containing neurons could be detected in the PVN and the lateral hypothalamus (and were identified as orexinergic), suggesting the migration and differentiation of progenitor cells from the ependymal layer. In this study, selected tanycyte/ependymal precursors formed neurospheres *in vitro* [64]. These neurospheres exhibited self-renewal and differentiated into a variety of cell types including neurons and glia [64]. Several subsequent studies have also demonstrated this capacity of hypothalamic neural stem cells (summarized in Table 1). The situation is complicated by the fact that tanycytes are not the only neural stem cells in the hypothalamus and that cells found in the mediobasal parenchyma also have this capacity [58,61–63].

Perhaps the most compelling evidence for hypothalamic neural stem cells comes from exploiting Cre–lox labeling strategies to trace the lineage of any new daughter cells [57,58]. In such studies, expression of Cre-recombinase was driven by an appropriate promoter (e.g., Sox2) in mouse strains carrying the yellow fluorescent protein (YFP) gene behind a loxP-flanked stop cassette in the ROSA26 locus. In neural stem cells expressing Sox2, the stop cassette is excised, allowing subsequent expression of YFP in all daughter cells. In these studies it is clear that generation of new cells with a variety of phenotypes occurs in the hypothalamus *in vivo*, confirming the existence of neural stem cells in the adult, of which tanycytes are a significant component [57,58] (Table 1).

### Relationship with feeding and energy balance

Why should neural stem cells exist in the adult hypothalamus and what might their functional roles be? One hypothesis is that they contribute to the plasticity and remodeling of the hypothalamic networks involved in the control of energy balance in a diet-responsive manner. Several studies are starting to make this link (Table 1). For example, ciliary neurotrophic factor (CNTF) triggers neurogenesis in the arcuate and ventromedial hypothalamus [61,62] and also causes weight loss. When division and proliferation of the stem cells (in this case only a small proportion were tanycytes) was prevented, the weight loss did not occur [61,62].

At the median eminence, β2 tanycytes increased their proliferation when mice were fed a high-fat diet. Cre-lineage tracing revealed labeled daughter neurons in the median eminence one month later [57]. These newly generated neurons may contribute to energy homeostasis because inhibition of tanycyte division and differentiation (by focused irradiation of the hypothalamus) reduced the accumulation of body weight in response to a high-fat diet. A contrasting study also utilized genetic manipulation of hypothalamic neural stem cells to demonstrate their importance for the control of feeding and energy balance [58]. This stem-cell population comprised cells from the parenchyma of the mediobasal hypothalamus as well as a smaller but still significant proportion of tanycytes (in this case, α and β1, but not β2 cells). By exploiting the sensitivity of these stem cells to IKKβ/NF-κβ signaling, the population of Sox2-positive cells in the hypothalamus was selectively reduced *in vivo*, and thus the capacity to generate new neurons was reduced. After 3 months, these mice exhibited impaired glucose tolerance and, after 10 months, they had become severely obese [58]. This finding complements other studies showing that a high-fat diet increases apoptosis of neurons in the arcuate and lateral hypothalamus [65].

Photoperiodic mammals also link α tanycyte proliferation to body weight (Table 1). Tanycytes express the enzymes that synthesize retinoic acid (i.e., RALDH1 and RALDH2) [44,45]. Interestingly, in F344 rats under long photoperiod, expression of RALDH1 in tanycytes was at its highest and the proliferation of the progenitor cells at its lowest [45]. The converse held under conditions of short photoperiod. Because retinoic acid inhibits proliferation of third ventricle progenitor cells [45], these findings provide strong evidence *in vivo* for retinoic acid signaling, acting via the proliferation of tanycytes, to contribute to the control of body weight.

A very tentative consensus from Table 1 is that, in those instances where the neural stem-cell population is comprised at least partially of α tanycytes, stem-cell proliferation is associated with either weight loss or the maintenance of a healthy body weight. For the other stem-cell types (i.e., β2 tanycytes, and other mediobasal hypothalamic cells), proliferation appears to be associated with gain of body weight. This suggests that the factors controlling proliferation and differentiation of specific hypothalamic neural stem-cell types, including tanycytes, are likely to be of high importance in understanding the control of body weight. For tanycytes, these signals include retinoic acid, circulating growth factors, hormones, and ATP released from the tanycytes themselves (Table 1, Box 2).

## Concluding remarks

From being enigmatic and mysterious cells, tanycytes are now emerging with an almost puzzling diversity of potential roles. They are clearly chemosensors that can sense the composition of the CSF in the third ventricle. Equally, they are the locus for changes in expression of genes linked to the natural control of body weight in a long-term seasonal context. They also form at least two distinct sets of adult stem cells, which can respond to growth factors, and the status of the hypothalamic networks controlling energy balance and diet. Interestingly, these potentially interlinked aspects of tanycyte physiology (Box 3) could have consequences for hypothalamic function across widely different temporal scales. The Ca^2+^ signaling in tanycytes evoked by metabolites and transmitters has the potential to evoke relatively rapid changes (i.e., on a timescale of seconds to minutes) in nearby neurons. The alteration of gene expression in tanycytes presumably acts on timescales of several hours to days (or longer), whereas the birth of new neurons and functional incorporation into hypothalamic circuits is likely to require several weeks to take effect. Understanding how these different functions fit together will be greatly aided by the development of new tanycyte-specific genetic tools to label, trace, and manipulate these multifunctional cells.

## Figures and Tables

**Figure I:**
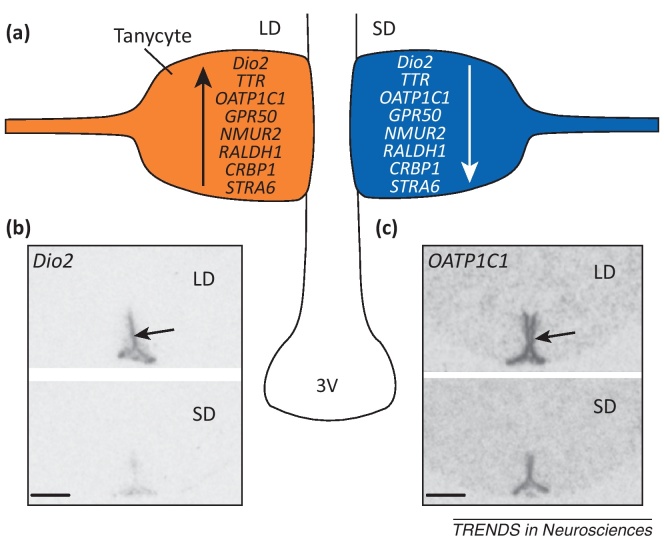
Gene expression in tanycytes changes with photoperiod. **(a)** Cartoon illustrating tanycytes under long and short photoperiod (LD and SD, respectively). The gene products regulating thyroid hormone signaling (i.e., Dio2 in hamster and rat [31,35–37], TTR and OATP1C1 in rat [31,42]), retinoic acid signaling (RALDH1, CRBP1, and STRA6 in rat [42]), as well as GPR50 (hamster) [23] and NMUR2 (rat) [42], are upregulated under LD compared to SD. **(b,c)** Examples of changes in mRNA levels for Dio2 and OATP1C1 in the F344 rat under LD (upper panels) and SD (lower panels). Representative autoradiographs showing, in coronal section, mRNA expression in the ependymal layer (arrows, containing the tanycytes) around the third ventricle (3V). Scale bars represent 1 mm. Adapted from [31].

**Figure I:**
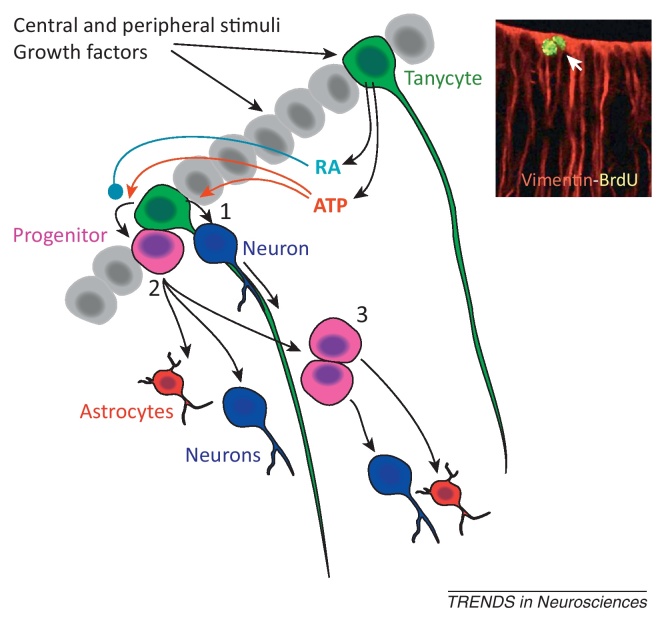
Proposed model for tanycyte proliferation. By analogy to radial glia, tanycytes could either generate new neurons directly (1), or via intermediate progenitor cells that could differentiate into neurons and/or astrocytes (2), or migrate as progenitors and divide further before differentiating (3). Inset: dividing vimentin-positive tanycytes revealed by BrdU staining [68]. These new neurons and progenitor cells could use the tanycyte processes as a means to migrate into the relevant hypothalamic nuclei. On this basis, tanycyte stem cells would form distinct populations to subserve the nuclei into which their processes project. Tanycytes are also capable of producing and releasing both retinoic acid (RA) [45] and ATP [11], which are proposed to act as inhibitors or activators of proliferation, respectively. The gray cells represent other tanycytes and are shaded merely for clarity of presentation. Inset adapted, with permission, from [68].

**Table 1:** Summary of the proliferating and neural stem-cell populations in the hypothalamus, the stimuli that induce proliferation, the evidence for their differentiation, and their link to changes in body weight^a^

Identity of hypothalamic cells	Stimulus for proliferation	Sox2^b^	Progenitor labeling method	Differentiation	Link to body weight	Age	Refs.
**Rat**							
Tanycytes (α)	bFGF and EGF	N/A	BrdU	Neurospheres, neurons in LH (orexin) and PVN, astrocytes	N/A	Adult	[64]
SD: stimulated LD: inhibited EGF: stimulated RA: inhibited	N/A	BrdU, Ki67	N/A	Proliferation associated with ↓ body weight	Adult	[45]
Tanycytes (α), sub-ependymal astrocytes	IGF-1	N/A	BrdU	Neurons (NeuN-positive)	N/A	2 months	[63]
Cells in ME, Arc, VMH, tanycytes (α and β), sub-ependymal astrocytes	Constitutive	N/A	BrdU	N/A	N/A	Adult	[68]
**Mouse**							
Cells in Arc, possibly a few α tanycytes.	Degeneration of NPY neurons	N/A	BrdU, Ki67, PCNA	Neurons: AgRP, POMC	Proliferation associated with maintenance of body weight	12 weeks	[69]
Cells in Arc, VMH and hypothalamic parenchyma and some tanycytes (α and β)	CNTF and constitutive	N/A	BrdU	Neurons: NPY, POMC; oligodendrocytes	Proliferation associated with ↓ body weight	Adult	[61]
Constitutive	N/A	BrdU, Ki67	Neurons in VMH	N/A	Adult	[62]
Tanycytes (β2), possibly other cells in parenchyma	Stimulated by acute HFD	+	BrdU, Cre lineage-tracing	Neurons in ME and some in Arc	Proliferation associated with ↑body weight	P35–P75	[57]
Tanycytes (α and β1, not β2), other cells in Arc and hypothalamic parenchyma	Inhibited by chronic HFD	+	BrdU, Cre lineage-tracing	Neurospheres, neurons, glia, oligodendrocytes	Loss of stem cells leads to obesity	Adult	[58]

a Abbreviations: AgRP, agouti-related peptide; Arc, arcuate nucleus; bFGF, basic fibroblast growth factor; EGF, epidermal growth factor; HFD, high-fat diet; IGF-1, insulin-like growth factor-1; LD, long photoperiod; ME, median eminence; N/A, not studied; PCNA, proliferating cell nuclear antigen; POMC, proopiomelanocortin; SD, short photoperiod; VMH, ventromedial hypothalamus.

b Sox2 is a defining marker for neural stem-cell populations [47,59,60].

## Box 1 Photoperiod-sensitive mammalian models

Mammalian models sensitive to photoperiod (e.g., Djungarian hamsters, the F344 strain of rat, and CBA/N and C3H strains of mouse) have unexpectedly started to link tanycytes with the control of body weight and energy balance. In the natural environment, seasonal alterations in daylength (i.e., photoperiod) induce behavioral and physiological adaptations such as hibernation, migration, changes in pelage, reproduction, and altered feeding. The seasonal variation of daylength is detected by the pineal gland and transduced via the secretion of melatonin, which occurs only during darkness. These photic clues allow animals to anticipate seasonal alterations in food availability and climate and to adapt by regulating energy balance. This type of regulation has been termed rheostasis [38], rather than homeostasis, because it involves longer-term adaptations to the set-points of adiposity and appetite. In broad terms, animals such as the Djungarian hamster and F344 rat, exposed to a short photoperiod (i.e., short days of the autumn and winter seasons) can be considered to be in a catabolic or ‘lean-like’ state, whereas those exposed to the long photoperiod (i.e., long days of the spring and summer) can be considered to be in an anabolic or ‘fat-like’ state. These two natural states provide a powerful method for examining differential patterns of gene expression related to body weight [31,38,66,67]. The surprising outcome of these studies is the identification of several genes expressed in tanycytes that exhibit photoperiodic regulation of expression (see main text for details and Figure I).

## Box 2 Hypothesis: tanycytes as a chemoresponsive stem-cell population

At least some tanycytes are stem cells and may also be responsive to diet, the lean/fat status of the body, and circulating factors. How do tanycytes ‘know’ when and where to generate new neurons? One possibility is that the chemosensitivity of tanycytes helps to determine whether they proliferate or not (Figure I). Tanycytes release ATP in response to glucose [11] which can act via P2Y1 receptors to stimulate Ca^2+^ waves. In other stem-cell populations, ATP-evoked Ca^2+^ waves act as a pro-proliferative signal [13,14]. Conversely, retinoic acid signaling in tanycytes decreases their ability to proliferate [45]. We therefore propose that the relative balance between these two pathways could be sensitive to a variety of external factors such as circulating hormones, metabolites, and growth factors. The integration of these signals by tanycytes could then determine whether they divide to generate new cells.

## Box 3 Outstanding questions

• **Are there functional subtypes of tanycytes?** Tanycytes are glucosensitive and respond to various peripheral and centrally generated signals. Tanycytes are also stem cells. Are all tanycytes capable of fulfilling both of these roles or are there specializations of function? Do all tanycytes respond to the same stimuli or are there subsets that are tuned to particular inputs?
• **What mechanisms control the proliferation of tanycytes?** How do tanycytes ‘know’ when to proliferate? How do they integrate the various peripheral and centrally generated inputs to ‘decide’ when and where to divide? Do specific tanycyte populations serve different hypothalamic nuclei as stem cells? Which types of neuron are generated from tanycytes? Are these neurons functionally active?
• **What is the purpose of tanycyte chemosensivity, and what are the underlying mechanisms?** Are tanycytes chemosensitive cells that sense the internal environment of the brain and pass this information onto hypothalamic neural networks to modulate their operation? Or is the purpose of this chemosensitivity primarily to regulate and control their own proliferation? What other stimuli activate tanycytes? What are the mechanisms of chemosensory transduction?
• **Do tanycytes communicate with neurons?** Tanycyte processes come very close to the glucosensing neurons of the ventromedical hypothalamus and the NPY-containing neurons of the arcuate nucleus [8,9]. This is at least suggestive of tanycyte-to-neuron signaling. Will a Ca^2+^ wave in a tanycyte cause the release of transmitters and consequently affect nearby neurons? If so, how, and which transmitters can tanycytes release? Which types of neurons can they influence? Are tanycytes inhibitory, excitatory, or both?
• **Do tanycytes control behavior?** Will persistent activation of tanycytes alter feeding behavior? Equally, what is the effect of persistent and selective inactivation of tanycytes? The effect of tanycytes on feeding behavior is hard to predict without knowing how they may influence hypothalamic networks.
